# Transforming Growth Factor-β and the Renin-Angiotensin System in Syndromic Thoracic Aortic Aneurysms: Implications for Treatment

**DOI:** 10.1007/s10557-020-07116-4

**Published:** 2020-12-07

**Authors:** Daan C.H. van Dorst, Nathalie P. de Wagenaar, Ingrid van der Pluijm, Jolien W. Roos-Hesselink, Jeroen Essers, A.H. Jan Danser

**Affiliations:** 1 Division of Vascular Medicine and Pharmacology, Department of Internal Medicine, Erasmus University Medical Center, Rotterdam, The Netherlands; 2 Department of Medical Oncology, Erasmus MC Cancer Institute, Erasmus University Medical Center, Rotterdam, The Netherlands; 3 Department of Molecular Genetics, Erasmus University Medical Center, Room Ee702b, Erasmus MC, Wytemaweg 80, 3015 CN Rotterdam, The Netherlands; 4 Department of Cardiology, Thoraxcenter, Erasmus University Medical Center, Rotterdam, The Netherlands; 5 Department of Vascular Surgery, Erasmus University Medical Center, Rotterdam, The Netherlands; 6 Department of Radiation Oncology, Erasmus University Medical Center, Rotterdam, The Netherlands

**Keywords:** Thoracic aortic aneurysm, Transforming growth factor-β, Renin-angiotensin system, Angiotensin receptor blockers, Marfan syndrome, Loeys-Dietz syndrome

## Abstract

Thoracic aortic aneurysms (TAAs) are permanent pathological dilatations of the thoracic aorta, which can lead to life-threatening complications, such as aortic dissection and rupture. TAAs frequently occur in a syndromic form in individuals with an underlying genetic predisposition, such as Marfan syndrome (MFS) and Loeys-Dietz syndrome (LDS). Increasing evidence supports an important role for transforming growth factor-β (TGF-β) and the renin-angiotensin system (RAS) in TAA pathology. Eventually, most patients with syndromic TAAs require surgical intervention, as the ability of present medical treatment to attenuate aneurysm growth is limited. Therefore, more effective medical treatment options are urgently needed. Numerous clinical trials investigated the therapeutic potential of angiotensin receptor blockers (ARBs) and β-blockers in patients suffering from syndromic TAAs. This review highlights the contribution of TGF-β signaling, RAS, and impaired mechanosensing abilities of aortic VSMCs in TAA formation. Furthermore, it critically discusses the most recent clinical evidence regarding the possible therapeutic benefit of ARBs and β-blockers in syndromic TAA patients and provides future research perspectives and therapeutic implications.

## Introduction: Thoracic Aortic Aneurysms

Thoracic aortic aneurysms (TAAs) are permanent pathologic dilatations of the thoracic aorta leading to an increase of more than 50% of the normal aortic diameter [1]. Although expansion of the aneurysm is asymptomatic, TAAs can suddenly lead to life-threatening complications, such as aortic dissection and aortic rupture [2]. In most TAAs, the risk of these possibly fatal events is directly proportional to the size of the aneurysm [3, 4]. Therefore, aneurysm diameter is strictly monitored in affected patients and medical therapy is initiated in an attempt to slow down TAA expansion. Most often, β-adrenergic blockers (β-blockers) are prescribed to decrease blood pressure, cardiac contractility and shear stress on the aorta, however at variable efficacy [5]. If the aneurysm diameter exceeds certain dimensions, timely prophylactic surgical or endovascular intervention is necessary. Generally, an aneurysm diameter between 50 and 55 mm is regarded as indication for intervention; however, some patients require surgery at smaller diameters depending on underlying conditions and hereditary factors [5]. Several risk factors for the development of TAAs have been identified, such as smoking and hypertension [1].

Although the majority of TAAs are sporadic, approximately 20% of TAAs occur in a syndromic or familial form in individuals with an underlying genetic predisposition [6, 7]. The most notable of these TAA syndromes are Marfan syndrome (MFS) and Loeys-Dietz syndrome (LDS), in which TAAs will develop in a high proportion of the affected individuals [8, 9]. Over the past 2 decades, both preclinical and clinical studies identified pathophysiological mechanisms that lead to TAAs, particularly in MFS patients. Substantial evidence demonstrates that increased signaling of the transforming growth factor-β (TGF-β) pathway plays an important role in thoracic aortic pathology [10, 11]. Nonetheless, the exact contribution of TGF-β signaling to TAA pathogenesis remains subject of debate [10, 12]. Additionally, the renin-angiotensin system (RAS) interacts with TGF-β signaling on multiple levels [13] and is also shown to constitute an important contributor to TAA formation [14, 15]. More recently, impairments in cellular responses to progressive hemodynamic aortic load (mechanosensing and mechanotransduction) in syndromic individuals have been put forward to predispose to hereditary TAAs [16, 17]. Multiple studies have investigated the therapeutic potential of compounds that inhibit TGF-β signaling or the RAS-TGF-β interaction in the context of TAAs, such as TGF-β neutralizing antibodies or angiotensin receptor blockers (ARBs). This review focuses on the role of TGF-β signaling and its interactions with RAS in syndromic TAA formation. Subsequently, the most recent clinical evidence concerning the usage of ARBs and β-blockers to attenuate aneurysm expansion is highlighted and critically discussed. Finally, future research perspectives and clinical implications are provided for affected TAA patients.

## The TGF-β Signaling Pathway and Its Role in TAA Development

TGF-β signaling plays a critical role in multiple biological processes, including cellular growth, differentiation, apoptosis, migration and extracellular matrix (ECM) production [18]. Three homologous TGF-β isoforms exist (TGF-β1, TGF-β2, and TGF-β3), which are produced by multiple cell types as precursor proteins [19]. Increasing evidence suggests different physiological roles of these isoforms and mutations in TGF-β2 and TGF-β3 have been associated with TAA formation [20–23]. However, most studies in the context of thoracic aortic pathology did not discriminate between these three isoforms and this review will therefore refer to these isoforms collectively as “TGF-β", unless stated otherwise. After intracellular cleavage, TGF-β remains associated with the cleaved part, the latency-associated peptide (LAP), through non-covalent interaction. Together they constitute the small latent complex (SLC). In turn, the latent TGF-β binding protein (LTBP) interacts with LAP to form a large latent complex (LLC) before being secreted by the cell [24]. After secretion, LTBPs interact with several constituents of the ECM, including fibrillin microfibrils [25]. In this way, TGF-β is sequestered in the ECM, which serves as an important TGF-β reservoir. Originally, fibrillin-1 was described as a structural component of the ECM [26], but more recently it was found that fibrillin-1 can also regulate the bioavailability of TGF-β1 by displacing the latency complex from microfibrils [27]. Besides, matrix metalloproteinases (MMPs), integrin presentation, reactive oxygen species, and thrombospondin-1 are involved in regulating release from the LLC [28–33].

**Fig. 1:**
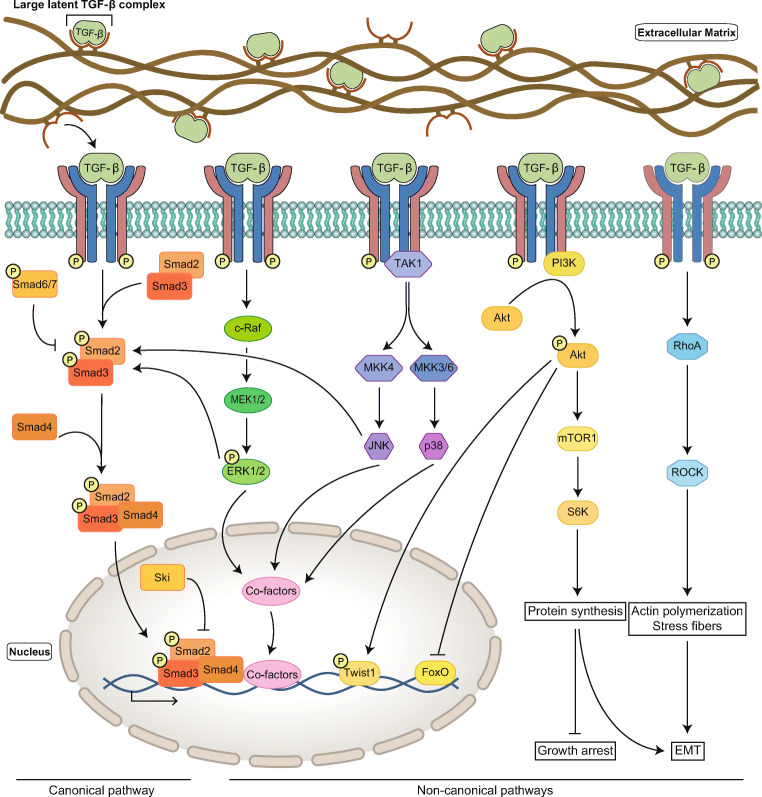
Overview of the TGF-β signaling pathway. After cellular secretion, TGF-β is sequestered in the ECM as the large latent complex (LLC). Upon release from the LLC, TGF-β binds, assembles, and activates the TGF-β receptor. Activation of this receptor complex initiates canonical and multiple non-canonical TGF-β signaling pathways. From left to right the canonical pathway, ERK1/2 pathway, JNK/p38 pathway, PI3K/Akt pathway, and small GTPase pathway

After its release and activation, TGF-β causes assembly of the TGF-β receptor complex, which consists of two TGF-β receptors I (TGFBR1) and two TGF-β receptors II (TGFBR2). Binding of active TGF-β to TGFBR2 recruits TGFBR1, which becomes activated through phosphorylation by the constitutively active TGFBR2-kinase domain. Subsequently, this signal is propagated to downstream intracellular signaling cascades, which can be divided into one canonical and multiple non-canonical pathways [34–36] (Fig. 1). Interestingly, both canonical and non-canonical signaling pathways have been shown to be involved in TAA development [37–40].

### Canonical TGF-β Pathway

The canonical TGF-β pathway is also known as the SMAD (Small Mothers Against Decapentaplegic)-dependent TGF-β pathway, in which the activated TGF-β receptor phosphorylates SMAD2 and SMAD3, the so-called receptor-regulated SMADs or R-SMADs (Fig. 1). Once phosphorylated, the R-SMADs have a high affinity to form a heterotrimeric complex with SMAD4 or co-SMAD. These SMAD complexes translocate to the nucleus where they interact with promotors/co-factors to regulate the transcription of downstream target genes [41–46]. These target genes are involved in the regulation of numerous processes, including ECM turnover, apoptosis and cellular proliferation, differentiation, motility and adhesion [10, 47]. Two inhibitory SMADs (I-SMADs), SMAD6 and SMAD7, are part of a negative feedback loop to regulate TGF-β signaling (Fig. 1). Both I-SMADs can inhibit TGF-β signaling and bone morphogenetic protein (BMP) signaling, although the effect of SMAD6 is modest [48, 49]. Increased TGF-β signaling correlates with increased expression of these I-SMADs, suggesting that their transcription is regulated downstream of the TGF-β pathway [50–52]. In addition, the SKI oncogene (SKI) protein inhibits the TGF-β pathway by repression of SMAD protein activity (Fig. 1) [53].

### Non-canonical TGF-β Pathways

TGF-β signaling additionally acts via four non-canonical (SMAD-independent) pathways: ERK1/2, JNK/p38, PI3K/Akt, and small GTPase [54] (Fig. 1). The first three of these pathways result in the activation of transcription factors that regulate apoptosis and epithelial-to-mesenchymal transition (EMT), often in concert with SMADs [54]. During EMT, epithelial cells differentiate into mesenchymal cells through the loss of polarity and cell-cell adhesions and acquire the capability to migrate, proliferate and differentiate. Although mesenchymal cells play a key role in tissue repair, they can also induce pathology by stimulation of tissue fibrosis [55]. Pathological EMT is predominantly associated with tumor metastasis, but this process also affects the integrity of the aortic ECM by promoting fibrosis and expression of MMPs, possibly contributing to TAA formation [36, 46]. The small GTPase pathway is involved in dissolution of tight junctions and cell adhesion. Furthermore, it stimulates the formation of membrane protrusion and induces, via Ras homology family member A (RhoA), actin polymerization and stress fiber formation. This again affects EMT in concert with SMAD signaling, as SMAD also stimulates RhoA [54].

### Crosstalk of Canonical TGF-β Pathway and Non-canonical TGF-β Pathways

Multiple modes of crosstalk between canonical and non-canonical TGF-β signaling can be distinguished. Both ERK1/2 and JNK are capable of phosphorylating R-SMADs to alter their activities, and the PI3K/Akt pathway can directly influence the net outcome of SMAD signaling [40, 54]. In turn, inhibitory SMADs have been demonstrated to regulate ERK and JNK/p38 signaling [56]. In this way, complex interactions are established, and their net outcomes are often highly dependent on the cellular context in which they occur. Next to direct interactions, another important crosstalk between canonical and non-canonical TGF-β signaling occurs at the level of DNA transcription. An example concerns the Smad3/4 complex and ERK, which have been shown to act as essential synergistic co-modulators of transcription of multiple TGF-β target genes. The extensive connections between the canonical and non-canonical TGF-β signaling pathways and the processes that are regulated by their crosstalk are reviewed in more detail elsewhere [56].

### TGF-β Pathway Genes Associated with Aneurysm Formation

TAAs are associated with a wide range of genetic mutations, which can broadly be classified into 3 main groups: genes encoding ECM constituents, genes involved in the contractile apparatus of vascular smooth muscle cells (VSMCs) and genes involved in the TGF-β pathway [57]. Table 1 gives an overview of important genetic defects leading to TAA formation and associated syndromes and phenotypes.

**Table 1:** Genetic defects causing aortic aneurysms in human

Gene	Protein	Chromosomal locus	Disease	Main clinical features	Reference
*FBN1*	Fibrillin-1	15q21.1	Marfan syndrome	Aortic root aneurysm and dissection, ectopia lentis, myopia, pectus deformity, arachnodactyly	[58, 59]
*BGN*	Biglycan	Xq28	Meester-Loeys syndrome	Early-onset aortic aneurysm and dissection, hypertelorism, pectus deformity, joint hypermobility, contractures, mild skeletal dysplasia	[64]
*EFEMP2*	EGF-containing fibulin-like extracellular matrix protein 2	11q13.1	Cutis laxa type 1B	Multiple arterial aneurysms and tortuosity, cutis laxa, joint laxity, arachnodactyly	[66, 67]
*TGFBR1*	TGF-beta receptor type-1	9q22.33	Loeys-Dietz syndrome type I	Widespread and aggressive arterial aneurysms and dissections, arterial tortuosity, hypertelorism, cleft palate, bifid uvula, pectus deformity, scoliosis	[68]
*TGFBR2*	TGF-beta receptor type-2	3p24.1	Loeys-Dietz syndrome type II	Widespread and aggressive arterial aneurysms and dissections, arterial tortuosity, hypertelorism, cleft palate, bifid uvula, pectus deformity, scoliosis	[68]
*SMAD3*	Small mothers against decapentaplegic homolog 3	15q22.33	Loeys-Dietz syndrome type III (also known as aneurysm-osteoarthritis syndrome)	Widespread and aggressive arterial aneurysms and dissections, arterial tortuosity, early-onset osteoarthritis, osteochondritis dissecans, hypertelorism, bifid uvula	[69, 70]
*TGFB2*	Transforming growth factor beta-2	1q41	Loeys-Dietz syndrome type IV	Thoracic aortic aneurysm and dissection, arterial tortuosity, mitral valve prolapse, arachnodactyly, flat feet, high arched palate, joint hyperflexibility	[21]
*TGFB3*	Transforming growth factor beta-3	14q24.3	Loeys-Dietz syndrome type V	Aortic aneurysm and dissection, mitral valve prolapse, hypertelorism, arachnodactyly, cleft palate, bifid uvula, pectus deformity, scoliosis	[22]
*SMAD2*	Small mothers against decapentaplegic homolog 2	18q21.1	Loeys-Dietz syndrome type VI	Arterial aneurysms and dissections, valve abnormalities, hypertelorism, pectus deformity, scoliosis, osteoarthritis, hernias	[71, 72]
*SMAD4*	Small mothers against decapentaplegic homolog 4	18q21.2	Haemorrhagic telangiectasia	Gastrointestinal hamartomatous polyps, cutaneous and mucosal telangiectasia, epistaxis, arteriovenous malformations	[77]
*SMAD6*	Small mothers against decapentaplegic homolog 6	15q22.31	Bicuspid aortic valve/thoracic aortic aneurysm	Bicuspid aortic valve, thoracic aortic aneurysm	[80]
*SKI*	Ski oncogene	1p36.33-p36.32	Shprintzen-Goldberg syndrome	Craniosynostosis, hypertelorism, high palate, micrognathia, arachnodactyly, joint contractures, pectus deformity, hypotonia, developmental delay	[78, 79]

The most notable in the first group are mutations in the *FBN1* gene, encoding the fibrillin-1 protein. Mutations in *FBN1* cause MFS, an autosomal dominant connective tissue disorder [58–60]. As fibrillin-1 is a principal component of the extracellular microfibrils, abnormal or deficient production of this protein distorts structural integrity of the ECM [61]. Besides, *FBN1* mutations are demonstrated to hamper TGF-β sequestration in the ECM, which leads to enhanced TGF-β bioavailability and increased activation of TGF-β signaling [27, 62]. Clinically, MFS is characterized by aortic aneurysms mainly located at the level of the aortic root and multiple cardiovascular, skeletal, and ocular manifestations, such as prolapse of the atrioventricular valves, anterior chest deformity, arachnodactyly, myopia, and ectopia lentis [8, 58, 59]. Importantly, aortic dissections occur in up to 70% of MFS patients and constitute the main cause of premature death [63]. Another prominent example of this first group of mutations are mutations in *BGN*, encoding biglycan, a small proteoglycan involved in assembly and maintenance of the ECM which is known to interact with TGF-β [64]. Lack of functional biglycan results in increased TGF-β bioavailability, mainly in the vascular adventitia layer which is the major site of biglycan deposition [65]. Patients with *BGN* mutations are characterized by early-onset aortic aneurysms and aortic dissections, hypertelorism, pectus deformity, joint hypermobility, contractures, and mild skeletal dysplasia [64]. Lastly, mutations in *EFEMP2* lead to impaired function of the EGF-containing fibulin-like extracellular matrix protein 2, better known as fibulin-4. Fibulin-4 interacts with multiple ECM components and is involved in elastogenesis, cross-linking, and regulation of TGF-β activity. Fibulin-4 mutations cause cutis laxa type 1B, which is a syndromic autosomal recessive condition characterized by aortic aneurysms, non-elastic loose skin (cutis laxa), vascular deformities, aneurysms, joint laxity and arachnodactyly [66, 67].

Examples of mutations in contractile apparatus genes leading to TAA are smooth muscle cell-specific isoforms of α-actin (*ACTA2*) and myosin heavy chain (*MYH11*). Mutations in these genes result in non-syndromic TAA and are reviewed elsewhere [16].

This review particularly focuses on genetic mutations affecting the TGF-β pathway that lead to TAA formation. In the past 2 decades, numerous mutations in genes encoding proteins of the TGF-β pathway were discovered to be associated with aneurysm formation. Notably, different mutations in the TGF-β pathway cause LDS, an autosomal dominant connective tissue disorder. LDS can be classified into six different subtypes, according to the underlying mutation (*TGFBR1*, *TGFBR2*, *SMAD3*, *TGFB2*, *TGFB3*, and *SMAD2*) [21, 22, 68–73]. LDS patients share many phenotypical characteristics with MFS patients, but LDS vascular pathology is often more aggressive and more widespread, affecting the entire arterial tree. This includes widespread arterial tortuosity and aneurysms of various medium-to-large muscular arteries, including the aorta and cerebral arteries [74]. Importantly, aortic aneurysms tend to dissect at smaller diameters and at a younger age [74, 75] and a high proportion of the affected individuals eventually require surgical intervention [76]. In addition, mutations in *SMAD4* and in *SKI*, an inhibitor of the TGF-β pathway, cause hemorrhagic telangiectasia and Shprintzen-Goldberg syndrome respectively. Both conditions are associated with thoracic aortic pathology, although this is less common than in MFS and LDS [77–79]. Besides, mutations in *SMAD6*, another inhibitor of TGF-β signaling, are associated with bicuspid aortic valve and TAA [80]. Altogether this highlights the essential role of the TGF-β pathway in cardiovascular diseases, particularly, but not limited to, the formation of TAAs.

### Mouse Models for TAA

The identification of genes responsible for heritable TAAs enabled the generation of mouse models with a TAA phenotype (Table 2). Of note, the exact TAA phenotypes vary considerably, due to differences in genetic background. Nonetheless, these mouse models allowed not only a more in-depth analysis of the effects of these different genetic mutations but also an evaluation of different potential treatment modalities targeting aneurysm growth.

**Table 2:** Genetic mouse models for thoracic aortic aneurysms

Gene	Mouse model	Expression	Phenotype	References
*Fbn1*	*Fbn1*^*mgN/mgN*^	No *Fbn1* expression	Postnatal death within 2 weeks due to ruptured aortic aneurysm, impaired pulmonary function, and/or diaphragmatic collapse, aneurysms mainly located in the ascending aorta	[81]
	*Fbn1*^*mgΔ/mgΔ*^	10-fold reduced *Fbn1* expression	Postnatal death within 3 weeks of age due to cardiovascular complications	[82]
	*Fbn1*^*GT-8/GT-8*^	No significant *Fbn1* expression	Postnatal death within 3 weeks of age	[83]
	*Fbn1*^*mgR/mgR*^	4- to 5-fold reduced *Fbn1* expression	Average lifespan of 4 months, aneurysm formation and dissection/rupture, pulmonary insufficiency	[84]
	*Fbn1*^*C1041G/+*^	Missense mutation, normal expression	Normal lifespan, aortic aneurysm formation	[85]
*Smad3*	*Smad3*^*−/−*^	No *Smad3* expression	Sudden death between 6 and 30 weeks of age due to thoracic aneurysmal dissection or cardiac tamponade, aneurysm formation in aorta and other vessels, increased aortic length, fragmentation of the elastic laminae, immune cell filtration	[39, 89]
*Tgfbr1*	*Tgfbr1* knock-out	No *Tgfbr1* expression	Embryonically lethal, severe defects in vascular development	[92]
	*Tgfbr1* conditional knock-out (endothelium, VSMCs and neural crest)	No *Tgfbr1* expression in specific cell types	Endothelium and VSMC specific: embryonically lethal, severe defects in vascular development Neural crest specific: lethal during birth or postnatal-hours, severe cardiovascular and pharyngeal defects	[90, 93]
	*Tgfbr1*^*M318R/+*^	Missense mutation, normal expression	Predisposition to aortic dissection and early death, aortic root aneurysm, tortuosity, elastic fiber fragmentation, craniofacial and skeletal abnormalities	[96, 97]
*Tgfbr2*	*Tgfbr2* knock-out	No *Tgfbr2* expression	Embryonically lethal, severe defects in vascular development	[91]
	*Tgfbr2* conditional knock-out (endothelium, VSMCs and neural crest)	No *Tgfbr2* expression in specific cell types	Endothelium and VSMC specific: embryonically lethal, severe defects in vascular development Neural crest specific: immediate postnatal death, severe cranial and cardiovascular malformations	[90, 94, 95]
	*Tgfbr2* postnatal SMC-specific deletion	95% reduction of *Tgfbr2* expression after tamoxifen treatment	Aortic dilatation, dissection, hemorrhage and ulceration, elastolysis, macrophage infiltration, abnormal proteoglycan accumulation	[98, 115]
	*Tgfbr2*^*G257W/+*^	Missense mutation, normal expression	Predisposition to aortic dissection and early death, aortic root aneurysm, tortuosity, elastic fiber fragmentation, craniofacial and skeletal abnormalities	[97]
*Fbln4*	*Fbln4*^*−/−*^	No *Fbln4* expression	Early postnatal death, arterial tortuosity, aortic aneurysm formation and rupture	[99]
	*Fbln4*^*f/−*^*/SM22Cre+*	No *Fbln4* expression in VSMCs	Reduced lifespan, aneurysm formation, fragmentation of the elastic laminae	[100–102]
	*Fbln4*^*R/R*^	4-fold reduced *Fbln4* expression	Sudden death within first 3 weeks of life, aneurysm formation and dissection	[103, 104]
	*Fbln4*^*E57K/E57K*^	Missense mutation, normal expression	Aneurysm formation, fragmentation of the elastic laminae	[67, 105]

Firstly, several mouse models for MFS exist, harboring mutations in *Fbn1*. *Fbn1*^*mgN/mgN*^ and *Fbn1*^*GT-8/GT-8*^ mice lack *Fbn1* expression, *Fbn1*^*mgΔ/mgΔ*^ mice have 10-fold reduced *Fbn1* expression and *Fbn1*^*mgR/mgR*^ have 4- to 5-fold reduced *Fbn1* expression [81–84]. Finally, *Fbn1*^*C1041G/+*^ mice mimic a patient missense mutation (C1039Y), which results in reduced formation of fibrillin-1 fibers, despite normal *Fbn1* expression [85]. Of note, *Fbn1*^*mgN/mgN*^, *Fbn1*^*GT-8/GT-8*^ mice, and *Fbn1*^*mgΔ/mgΔ*^ mice die in the first weeks of life, whereas homozygous *Fbn1*^*mgR/mgR*^ mice die around 4–6 months due to aortic dissections [81–84]. Although MFS patients nowadays have near-normal lifespan [86], the average life expectancy for MFS patients in 1972, before the evolution of open-heart surgery, was reported to be 32 years and most patients died due to aortic dissections [87, 88]. This phenotype is mostly phenocopied by untreated *Fbn1*^*mgR/mgR*^ mice. In contrast, *Fbn1*^*C1041G/+*^ mice have a normal life span [85]. This could imply that lower expression of *Fbn1* results in a more severe phenotype.

Secondly, several mouse models for LDS have been established. Full knock-out models for *Tgfbr1*, *Tgfbr2* and *Smad3* were generated, as well as mice with a conditional knock-out of *Tgfbr1* and *Tgfbr2* specifically in the endothelium, VSMCs or neural crest [39, 89–95]. In addition, a mouse model with a postnatal SMC-specific *Tgfbr2* deletion and *Tgfbr1*^*M318R/+*^ and *Tgfbr2*^*G257W/+*^ mouse models with heterozygous kinase-inactivating missense mutations were created [96–98]. *Smad3* knock-out mice develop aneurysms and histological analysis of TAA tissue reveal fragmentation of the elastic laminae and aortic immune infiltration, similar to the alterations observed in the human situation [39, 89]. Conventional knock-outs for *Tgfbr1* or *Tgfbr2* and conditional knock-outs for *Tgfbr1* or *Tgfbr2* in endothelial cells or VSMCs are embryonic lethal, due to serious defects in vascular development [90–92]. Conditional knock-out of *Tgfbr1* or *Tgfbr2* is lethal during birth or in the immediate postnatal period [93–95]. Postnatal SMC-specific *Tgfbr2* deletion results in vascular abnormalities, which are also found in LDS patients [98]. *Tgfbr1*^*M318R/+*^ and *Tgfbr2*^*G257W/+*^ mice have cardiovascular and craniofacial abnormalities, partly overlapping with the characteristics of LDS patients [96, 97].

Thirdly, several mouse models harboring mutations in *Fbln4*, encoding fibulin-4, have been created. As mentioned, fibulin-4 plays an important role in elastic fiber formation and mutations in *EFEMP2* (the human gene encoding fibulin-4) cause cutis laxa type 1b. Importantly, a complete knock-out of *Fbln4 (Fbln4*^*−/−*^) results in perinatal death [99]. In order to generate a more viable mouse model, a VSMC-specific knock-out (*Fbln4*^*f/−*^*/SM22Cre+*) was generated, in which aneurysms in the ascending aorta develop rapidly, accompanied by fragmentation of the elastic laminae [100–102]. Besides, *Fbln4*^*R/R*^ mice, expressing only 25% of fibulin-4 compared to wild-type mice, present with aneurysms throughout the entire aorta, fragmentation of the elastic lamina, and increased ECM deposition. In these mice, increased upstream and downstream TGF-β signaling was detected by elevated levels of TGF-β ligand, increased SMAD2 phosphorylation and increased activation of the downstream target plasminogen activator inhibitor-1 (PAI-1) [103, 104]. Lastly, *Fbln4*^*E57K/E57K*^ mice express a mutated form of fibulin-4, which impairs binding to LTBP1 and reduces assembly of the ECM. Similarly, this results in aneurysm formation and fragmentation of elastic laminae [67, 105].

### The Role of TGF-β Signaling in TAA

Over the years, dysregulation of TGF-β signaling has been proposed in multiple TAA syndromes. Neptune et al. were the first to propose a detrimental role of TGF-β signaling in MFS, as they demonstrated that mice deficient in fibrillin-1 (*Fbn1*^*mgΔ/mgΔ*^) have upregulated TGF-β activation and signaling. This led to impaired lung development due to increased cellular apoptosis [27]. As these *Fbn1*^*mgΔ/mgΔ*^ mice die around postnatal day 12, a *Fbn1* mouse model with a missense mutation (*Fbn1*^*C1041G/+*^) was created to enable better analysis of aneurysm development. In this model, Habashi et al. demonstrated that increased TGF-β signaling, based on increased pSMAD2 levels, is associated with TAA development [106]. Additional evidence for the involvement of TGF-β signaling in TAA was the finding that mutations in TGF-β pathway genes also predispose to TAA, such as in LDS patients (Table 1). Remarkably, most of these mutations constitute loss-of-function mutations, but yet seem to enhance TGF-β signaling [107]. Indeed, nuclear accumulation of phosphorylated SMAD2 and SMAD3 and increased expression of TGF-β-regulated genes was found in LDS patients with *TGFBR1* and *TGFBR2* mutations. This is indicative of upregulated TGF-β signaling [68, 108, 109]. Indeed, pSMAD2 activation is often used as a readout for TGF-β activation [22, 97, 106]. Interestingly, upregulated TGF-β signaling is also observed with mutations in the other genes that cause LDS, such as *TGFB2*, *TGFB3*, and *SMAD3* [21, 22, 70]. How this paradoxically elevated TGF-β signaling, known as “the TGF-β paradox", is mediated at a functional level is still not fully understood. One potential explanation for the TGF-β paradox, observed in in *Smad3*^*−/−*^ mice, is that these genetic mutations lead to increased upstream canonical TGF-β signaling (pSMAD2), without affecting downstream canonical TGF-β signaling [39]. In addition, mutations in *FBN1* and *EFEMP2* were found to indirectly affect TGF-β signaling [103, 110]. In turn, excessive TGF-β signaling in adult aorta has been linked to improper remodeling of the vascular ECM, VSMC dysfunction and TAA development [111, 112]. Excessive TGF-β signaling also leads to increased expression and activity of MMPs, degradation of elastic fibers, enhanced proliferation and migration of VSMCs and excessive secretion and deposition of collagens [111, 113]. These effects could all contribute to weakening of the aortic wall, rendering it more vulnerable for dilation, dissection and rupture [111].

Interestingly, treatment with TGF-β-neutralizing antibodies, from 7 to 15 weeks of age, normalized TGF-β signaling in the aortic media and improved elastic fiber fragmentation and aortic wall architecture in *Fbn1*^*C1041G/+*^ mice. Also, this treatment reduced aortic wall thickness and attenuated growth of the aortic root [106]. This indicates that excessive TGF-β signaling contributes to the formation of aortic aneurysms and that TGF-β antagonism is a potential treatment strategy. Importantly, it has been shown that adequate timing of TGF-β neutralization is essential. In *Fbn1*^*mgR/mgR*^ mice, a beneficial effect of TGF-β neutralization was observed when the treatment was started after onset of aneurysm formation (8-week old mice). However, if treatment with TGF-β-neutralizing antibodies was started before onset of aneurysm formation (postnatal day 16), this led to an acceleration in aneurysm development [114]. Besides, prenatal VSMC-specific *Tgfbr2* deletion in mice results in defective elastogenesis, vessel wall dilatation, aneurysms, and dissections [94, 115, 116]. In contrast, these harmful effects did not occur when this VSMC-specific *Tgfbr2* deletion was induced in mice at the age of 8-weeks [115]. This indicates that TGF-β has a protective role against aneurysm formation early in vascular development. Indeed, the TGF-β signaling pathway has been demonstrated to play an essential role in the initial vascular formation during embryogenesis, as mice with a knock-out mutation in either *Tgfb1*, *Tgfbr1*, or *Tgfbr2* developed severe defects in yolk sac vasculogenesis and hematopoiesis [117], predisposing to early embryonic lethality [90]. Also, TGF-β signaling is important for the formation of the embryonic vascular plexus, proper elastogenesis during embryogenesis and during the first weeks of life and for the development, morphogenesis and migration of endothelial cells and VSMCs [111, 118, 119]. Once these processes are completed, TGF-β seems to switch to become a detrimental contributor to aortic pathology [106, 111, 114], as reflected by the observed beneficial effects of TGF-β neutralization in a later stage [106]. Thus, adequate timing of TGF-β neutralization seems essential to optimally benefit from its protective effects on TAA formation, sparing the early protective role of TGF-β signaling [114]. However, the consequences of TGF-β neutralization on aortic pathology also seems to be dependent on the animal model studied. In a mouse model in which aortic aneurysm formation was induced by continuous administration of angiotensin II (Ang II), concomitant treatment with a TGF-β neutralizing antibody promoted aortic rupture, mostly at the level of the ascending aorta and suprarenal aortic region. In contrast, treatment with a TGF-β neutralizing antibody after the Ang II treatment period had no detrimental effects on aortic morphology [120]. This again stresses the variable effects of TGF-β neutralization at different developmental timepoints.

Another observation that adds to the complexity of the exact role of TGF-β in aneurysm formation was described by MacFarlane et al. In their study, they found that the effect of the *Tgfbr1* mutation, and subsequent disturbance in TGF-β signaling in the *Tgfbr1*^*M318R/+*^ LDS mouse model, varies between VSMCs that are derived from distinct lineages of origin. Their study suggests that the predisposition to aortic root aneurysm in LDS mice depends on both defective SMAD signaling in secondary heart field–derived VSMCs and excessive SMAD signaling in cardiac neural crest-derived VSMCs [96]. This is in line with previous findings in avian systems, which show that neural crest– and mesoderm-derived VSMCs respond differently to TGF-β1. To be more precise, cellular proliferation and fibrosis were seen in the neural crest-derived VSMCs and growth inhibition in the mesoderm-derived VSMCs [106, 121, 122]. Although this concept has not been further verified in other aortic aneurysm syndromes, perturbations in TGF-β signaling could thus have different effects on the various cell types that comprise the aortic tissue.

In summary, combinatory evidence from TAA mouse models and human genetic studies reveals a dimorphic role of TGF-β signaling in the development of TAA. During embryogenesis and in the first weeks of life, it has protective effects, whereas at a later time point it is an important contributor to thoracic aortic pathology. Also, the exact effects of TGF-β signaling are suggested to depend on the developmental origin of aortic VSMCs.

## The Renin-Angiotensin System and Its Interaction with the TGF-β Signaling Pathway in Thoracic Aneurysm Formation

**Fig. 2:**
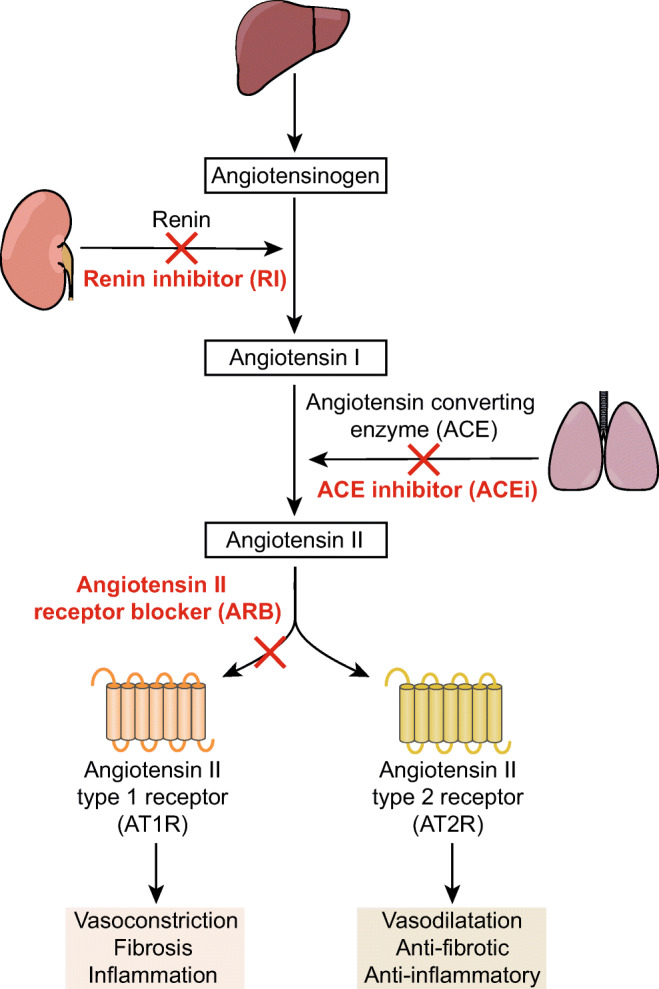
The renin-angiotensin system (RAS) and RAS blocking agents. Kidney-derived renin cleaves liver-derived angiotensinogen to generate angiotensin I, which is subsequently converted by angiotensin-converting enzyme (ACE) to angiotensin II, the main effector of the RAS. Angiotensin II stimulates its type 1 and 2 receptors, resulting in opposing effects on vasoactivity, fibrosis, and inflammation [14, 15]. RAS inhibition is possible with renin inhibitors, ACE inhibitors, and angiotensin II type 1 receptor blockers

**Fig. 3:**
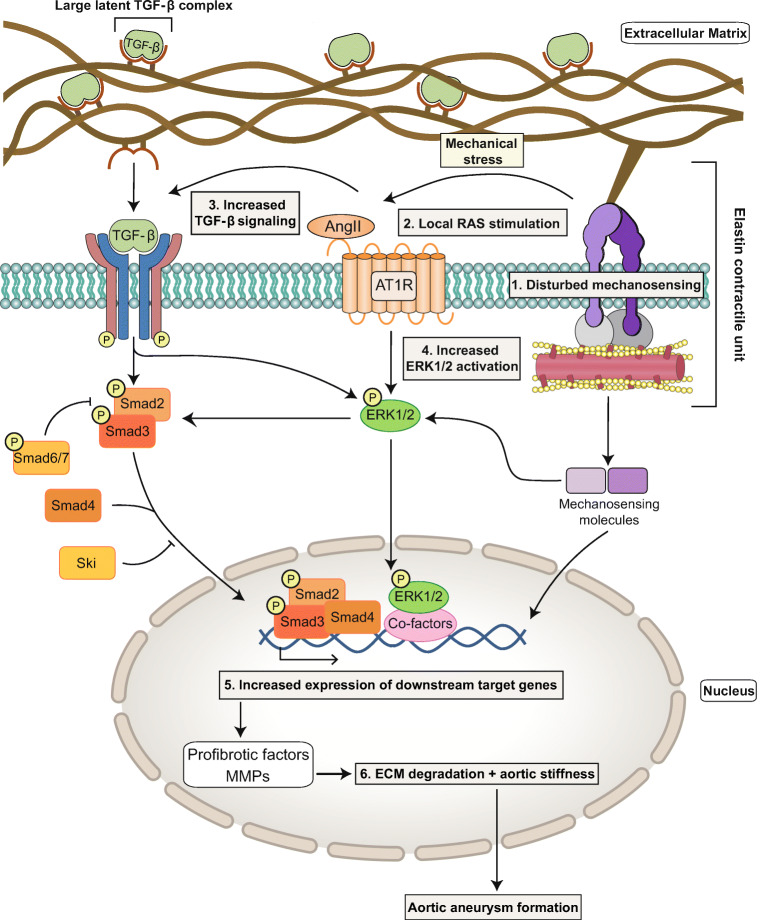
Hypothesized mechanism involving TGF-β signaling, RAS and disturbed mechanosensing abilities of aortic VSMCs that leads to TAA in syndromic individuals. Normally, TGF-β signaling, RAS, and mechanosensing in aortic VSMCs, via elastin-contractile units, cooperate to control aortic ECM remodeling and to maintain ECM integrity. However, in the situation of syndromic aneurysmal disease involving mutations in constituents of the ECM or the elastin-contractile unit, mechanosensing abilities are disturbed (1). This leads to mechanical stress, which stimulates local RAS activity by inducing ACE expression and activating the AT1R (2). Also, alternative, less-characterized mechanisms that stimulate local RAS activity might come into play in TAA formation. This increased RAS signaling can act as an upstream activator of TGF-β signaling (3) but also directly increase ERK1/2 signaling (4). In turn, TGF-β hyperactivity induces both canonical TGF-β signaling and non-canonical ERK1/2 signaling. Consequently, expression of target genes, among them MMPs and profibrotic factors such as connective tissue growth factor (CTGF) and PAI-1 is highly increased (5). Eventually, overexpression of these factors can lead to detrimental ECM degradation and aortic stiffness (6), which contributes to aortic aneurysm formation in affected syndromic individuals. In turn, ECM degradation leads to higher bioavailability of sequestered TGF-β, further amplifying this process

Another system that has repeatedly been reported to play an essential role in TAAs is RAS, which is a critical regulator of blood pressure, extracellular fluid homeostasis and electrolyte balance (Fig. 2). Ang II, the principal effector of the RAS, can stimulate both the Ang II type 1 receptor (AT1R) and the Ang II type 2 receptor (AT2R), but mainly acts via the former to exert its well-known functions, such as vasoconstriction, fibrosis and inflammation [14, 15]. Notably, infusion of Ang II leads to aneurysm formation of the abdominal aorta in apolipoprotein E-deficient mice [123, 124], but also to rapid expansion of the thoracic aorta with typical histological alterations, such as elastin fragmentation and increased medial thickness [123, 125, 126]. Additionally, increased plasma Ang II concentrations are observed in TAA patients [127, 128]. Interestingly, blockade of Ang II-AT1R signaling attenuated aortic root expansion in multiple preclinical and clinical studies [37, 106, 129, 130]. This stresses the importance of RAS in thoracic aortic pathology. Figure 3 summarizes several mechanisms by which the RAS may influence aneurysmal disease, which is discussed in more detail below.

### AT1R Signaling

As stated, Ang II mainly exerts its effects via the AT1R, which could contribute to TAA formation. An important aspect is also where exactly the vascular AT1Rs relevant for signaling in TAAs are located. Unlike humans, rodents have 2 types of AT1R (a and b). VSMC-specific deletion of the most abundant subtype (AT1aR) did not affect aortic pathology in *Fbn1*^*mgR/mgR*^ mice [131], nor in an ascending aortic aneurysm model (LDL receptor^−/−^ mice fed a saturated fat diet and infused with Ang II) [132]. Thus, either the relevant AT1R are located on other vascular cells (endothelial cells and/or fibroblasts) [131–133], or continued AT1R stimulation via remaining AT1bR might occur. In case of the former, a likely scenario is that VSMCs are affected by factors released from other vascular cells following their stimulation by Ang II. To rule out the latter, mice lacking both AT1R subtypes in VSMCs need to be studied.

### AT2R Signaling

AT2R stimulation is generally regarded to counter-regulate the effects mediated by the AT1R [15]. One of the rationales behind the therapeutic usage of ARBs is that the upregulated Ang II during RAS blockade is redirected from the blocked AT1R to the non-blocked AT2R to activate alternative transcriptional programs. This yields protection in numerous cardiovascular conditions, such as hypertension and heart failure. Yet, the exact role of the AT2R in aortic aneurysm remains controversial [134]. On the one hand, previous studies demonstrated that the ability of the ARB losartan to attenuate aortic enlargement required availability of AT2R signaling [135]. Also, knock-out of the AT2R gene resulted in larger aortic diameters and an increased number of premature deaths in *Fbn1*^*C1041G/+*^ mice [37]. As the exact causes of deaths in this study were not provided, it remains uncertain if these could be attributed to aortic complications, such as aortic dissection and rupture. In addition, renin inhibitors (RI) and angiotensin-converting enzyme inhibitors (ACEi), blocking both AT1R and AT2R signaling (Fig. 2), were less effective than ARBs in decreasing aortic root dilatation in *Fbln4*^*R/R*^ [136] and *Fbn1*^*C1041G/+*^ mice [37] and preventing transverse aortic constriction-induced aneurysm formation in wild-type mice [135]. Of note, only single dosages of these classes of drugs were investigated and other dosages might have yielded different outcomes. Nonetheless, these results advocate a protective role of the AT2R in aneurysm formation. On the other hand, a recent study demonstrated that direct AT2R stimulation with the AT2R agonist C21 did not show beneficial effects on TAAs in *Fbn1*^*C1041G/+*^ mice [137]. In line with this, blockade of the AT2R did not accelerate Ang II-induced aneurysm growth in hypercholesterolemic mice [123]. Lastly, losartan continued to exert beneficial effects on aortic pathology after deletion of the AT2R in mice harboring a VSMC-specific knock-out of *Fbln4* [100]. This indicates that AT2R activation in combination with AT1R blockade is only essential to yield protective effects under specific genetic conditions. Of note, the animal models in these studies display wide heterogeneity and the role of the AT2R in aortic pathology could depend on the exact genetic background, the mechanism by which the aneurysm is induced and timing and duration of treatment. In the human situation, most tissues display low AT2R expression, except during embryonic development [138]. Although AT2R upregulation is reported under pathological conditions, which could include aortic diseases [139], it is also possible that AT2R signaling comes into play at a later stage of aortic aneurysm development and is not involved in its initial pathogenesis [136].

### Interaction with TGF-β Signaling

The RAS interacts with TGF-β signaling at multiple levels and this might compose a mechanism by which it contributes to thoracic aortic pathology. Importantly, Ang II-AT1R signaling acts as an upstream activator of TGF-β signaling: Ang II directly increases TGF-β mRNA production, TGF-β protein expression and TGF-β activity [13, 140, 141]. As various TAA syndromes such as MFS and LDS are characterized by increased TGF-β signaling, this could be secondary to elevated RAS signaling. Indeed, abnormal upregulation of ACE and subsequent elevated local Ang II-AT1R signaling has been demonstrated in mice harboring a VSMC-specific knock-out of *Fbln4*, leading to ascending aortic aneurysms [100]. Also, enhanced AT1R downstream responses to Ang II stimulation are observed in *Fbln4*-deficient VSMCs [17]. Theoretically, increased RAS signaling in the context of aortic aneurysms could also be due to increased receptor density of the AT1R. However, to the best of our knowledge, this has not been studied in human TAAs yet.

### Perturbed Mechanosensing

One of the mechanisms that could lead to increased Ang II-AT1R signaling in syndromic TAAs is mechanical stress due to perturbations in mechanosensing. The aortic wall is continuously subjected to varying hemodynamic loads, such as a pulsatile blood pressure [142]. Normally, these mechanical forces are sensed by aortic VSMCs via elastin-contractile units (Fig. 3), which are composed of intracellular contractile elements linked to elastic fibers and microfibrils in the aortic ECM via focal adhesions [143]. In response to these mechanical cues, VSMCs activate downstream targets (mechanosensing molecules) which alter downstream gene expression to influence the composition of the ECM. Sensing the biomechanical environment (mechanosensing) and propagation of this signal intracellularly (mechanotransduction) to remodel the ECM under various hemodynamic circumstances are essential to ensure sufficient strength and compliance of the aortic wall and to maintain aortic wall integrity [143]. Consequently, genetic mutations in components of the elastin-contractile units, such as fibrillin-1, fibulin-4, and α-actin, lead to perturbed mechanosensing and result in inadequate ECM remodeling and mechanical stress. This predisposes to aortic aneurysms [17, 60, 101, 144] (Fig. 3). In addition, these abnormalities in mechanosensing are paralleled by elevated local ACE expression and increased Ang II signaling [100]. This could be a consequence of the resulting mechanical stress, which has been demonstrated to upregulate local ACE expression [145] and to even directly stimulate the AT1R, in an Ang II-independent manner [146, 147]. Therefore, it is most likely that elevated AT1R signaling in TAAs can best be prevented by ARBs and not by ACEi, as the latter can only prevent Ang II formation and not Ang II-independent AT1R signaling.

Abnormalities in mechanosensing lead to increased expression of mechanosensing molecules, which could further contribute to elevated TGF-β signaling, as observed in syndromic aortic aneurysms [17, 68, 148–150]. For instance, the mechanosensing molecule early growth response 1 (EGR1) has been shown to increase TGF-β gene expression by binding to its promotor and activating its transcription. Also, EGR1 induces expression of thrombospondin (*Thbs1*), another mechanosensing molecule that disturbs arterial wall integrity, activates ERK1/2 and latent TGF-β, and serves as an important mediator of Ang II-induced activation of TGF-β in rat cardiac fibroblasts and mesangial cells [13, 17]. Notably, deletion of *Thbs1* in mice harboring a VSMC-specific knock-out of *Fbln4* largely prevented TAA formation [17]. Thus, abnormal mechanosensing could contribute to aortic aneurysms by (1) hampering adequate ECM remodeling in response to hemodynamic loads, and (2) stimulation of local AT1R and TGF-β signaling pathways, via mechanisms that involve mechanical stress and pathological upregulation of mechanosensing molecules (Fig. 3). Nonetheless, much of these mechanisms remain only partly understood and require further research.

### ERK1/2 Signaling: Crosstalk Between RAS and TGF-β

An important question that remains is how elevated Ang II-ERK1/2 and TGF-β signaling drive aneurysmal disease. The mitogen-activated protein kinase (MAPK) signaling pathway forms an important crosstalk between RAS and TGF-β signaling: both Ang II and TGF-β, via non-canonical signaling, activate the 3 major MAP kinases: ERK1/2, JNK and p38 [36, 127]. Of these kinases, particularly ERK1/2 seems to be involved in aortic aneurysm formation, as the MAPK kinase (MEK) 1/2 inhibitor RDEA119 attenuated aortic root growth by selectively reducing ERK1/2 activation in *Fbn1*^*C1041G+*^ mice, without affecting JNK and p38 activity [38]. In this study, ERK1/2-blockade was highly effective, as aortic root growth in this group was even indistinguishable from wild-type mice. Additionally, accumulation of phosphorylated ERK1/2 (pERK1/2), the active form of ERK1/2, was associated with aneurysm growth in *Fbn1*^*C1041G+*^ mice, and both a TGF-β neutralizing antibody and losartan were able to reduce pERK1/2 levels and to attenuate aneurysm expansion [37]. Notably, the MAPK pathway has also been shown to directly induce canonical TGF-β signaling by phosphorylation and subsequent activation of R-SMAD proteins [36, 151], thereby forming a positive feedback loop (Fig. 3).

TGF-β signaling induces expression of MMPs, such as *MMP2* and *MMP9*, in aortic VSMC [127, 152–154], predominantly via non-canonical signaling pathways including ERK1/2 [155]. MMPs are involved in the degradation of ECM components and their activity is normally balanced by antagonizing tissue inhibitors of MMPs (TIMPs) to strictly control ECM turnover. Elevated TGF-β- and ERK1/2-induced MMP expression results in increased remodeling and incremental degradation of aortic connective tissue, which leads to weakness of the aortic wall. Consequently, the aortic capacity to endure biomechanical forces is reduced, which predisposes to aneurysm development [57, 153]. Indeed, a signature of elevated MMP expression is found in tissue samples from TAA patients [153, 156]. MMP inhibition could therefore have therapeutic potential, as evidenced by the observation that the MMP inhibitor doxycycline was more potent in preventing TAA and to preserve elastic fiber integrity than the β-blocker atenolol in *Fbn1*^*C1041G+*^ mice [157]. The beneficial effects of doxycycline on thoracic aortic pathology were also observed in other MFS mouse models (*Fbn1*^*C1041G/+*^ and *Fbn1*^*mgR/mgR*^) [158, 159]. However, MMPs do not seem to contribute to TAAs under all circumstances, as another preclinical study demonstrated that MMP2-deficiency actually accelerated dilatation of the thoracic aorta during Ang II infusion. Although this model may not fully mimic syndromic TAA patients, it demonstrates that a balance between Ang II and MMPs is essential to maintain ECM homeostasis. Another mechanism by which MMPs could contribute to aneurysm formation is by further increasing TGF-β signaling [30]. This is evidenced by the observation that activation of TGF-β and subsequent activation of the pSMAD2/3 pathway was hampered in *MMP2*-deficient mice [160]. Besides, TGF-β is normally sequestered in the ECM and increased ECM degradation mediated by MMPs could result in a greater TGF-β bioavailability [62]. Next to increased MMP expression, both AT1R signaling and TGF-β increase genetic expression of several profibrotic factors, such as connective tissue growth factor and plasminogen activator inhibitor-1 [161]. These factors may further impair aortic wall homeostasis by increasing aortic wall stiffness [161], which is an independent predictor of progressive aortic dilatation in MFS patients [162].

As stated, blockade of TGF-β signaling too early exacerbates aortic pathology [114]. Therefore, a better approach might be to specifically inhibit Ang II-AT1R-driven TGF-β overactivation, rather than TGF-β signaling in general. ARBs antagonize TGF-β overexpression in mice with chronic renal insufficiency and cardiomyopathy [163, 164]. Additionally, mechanical stress from disruptions in mechanosensing could theoretically be reduced by drugs that decrease hemodynamic load, such as β-blockers or alternative antihypertensive drugs.

## Clinical Trials in Syndromic TAA Patients

Over the past 2 decades, multiple randomized controlled trials have been performed to investigate the potency of ARBs and β-blockers to limit aortic aneurysm growth in the clinical situation (Table 3). Of note, all studies were performed in MFS patients and most trials used aortic (root) growth as a surrogate outcome for aneurysm rupture or dissection.

**Table 3:** Randomized controlled trials comparing angiotensin receptor blockers and β-blockers in Marfan syndrome patients

Trial name/principal investigator	Treatment arms	Mean enrollment age (years)	N	Baseline aortic root Z-score	Follow-up (months)	Primary outcome	Results	*P* value	Notes
COMPARE [129]	Losartan vs. standard care	36.8 and 38.3	233	3.9 vs. 3.8	36	Change in aortic diameter	+ 0.77 mm vs. + 1.35 mm/3 years	0.014	70% and 75% received β-blocker; open-label
Chiu, H [166]	Losartan + β-blocker vs. β-blocker	12.5 and 13.7	28	3.8 vs. 3.3	35	Change in aortic diameter	+ 0.10 mm vs. + 0.89 mm/year	0.02	Differences in baseline aortic Z-scores; open-label
Pediatric Heart Network Trial [167]	Losartan vs. atenolol	11.0 and 11.5	608	4.2 vs. 4.4	36	Change in aortic root Z-score	− 0.107 *z* vs. − 0.139 *z*/year	0.08	None
Marfan Sartan [168]	Losartan vs. placebo	30.9 and 28.9	299	3.7 vs. 3.7	42	Change in aortic root Z-score	− 0.03 *z* vs. − 0.01 *z*/year	0.68	86% received β-blocker in both groups
Forteza, A [169]	Losartan vs. atenolol	26.1 and 24.3	140	3.2 vs. 3.1	36	Change in aortic diameter and aortic root Z-score	+ 1.1 vs. + 1.4 mm/3 year and − 0.4 *z* vs. − 0.1 *z*/3 year	0.38 and 0.19	None
Muiño-Mosquera, L [170]	Losartan vs. placebo	35.4 and 36.8	22	3.6 vs. 3.5	36	Change in aortic root diameter and Z-score	+ 1 mm vs. + 1 mm/3 year and + 0.21 *z* vs. + 0.14 *z*/3 year	> 0.99 and 0.859	Underpowered study; high proportions of previous aortic root replacement
AIMS [130]	Irbesartan vs. placebo	18 (median)	192	3.2 vs. 3.3	60	Change in aortic root diameter and Z-score	+ 0.53 mm vs. + 0.74 mm/year and + 0.05 *z* vs. + 0.15 *z*/year	0.030 and 0.035	54% and 59% received β-blocker; losartan group had slightly lower BP throughout study
Di Toro, A [171]	Losartan + nebivolol vs. losartan MT or nebivolol MT	1–55	236	Unpublished	48	Differences in aortic root Z-score changes	Difference of − 0.16 *z* and − 0.18 *z*/2 years	0.019 and 0.032	Abstract only; open label

*Abbreviations*: *BP*, blood pressure; *MT*, monotherapy; *N*, numbers enrolled

Brooke et al. [165] were the first to examine the clinical benefits of ARBs on TAA pathology. In a retrospective study, they demonstrated that ARB usage was associated with reduced aortic root growth in severely affected pediatric MFS patients. Multiple randomized controlled trials followed [129, 130, 166–171] (Table 3). In 2013, the randomized open-label controlled COMPARE trial was published, which investigated the potential benefit of adding losartan to standard care, including a β-blocker in > 70% of the patients [129]. After 3 years of follow-up, aortic root aneurysm growth was significantly lower in the losartan group vs. the standard care group, confirming the previous retrospective results [165]. Although mean arterial blood pressure was slightly lower in the losartan group, no correlation between blood pressure levels and aortic root dimensions was found. This suggests that the beneficial effects of losartan are, at least partially, independent of its effects on blood pressure. This might be attributed to its inhibitory effects on TGF-β and ERK1/2-signaling, but clinical measurements to validate this hypothesis are lacking.

### ARBs vs. β-Blockers

Several trials compared the efficacy of ARB monotherapy with β-blocker monotherapy to prevent aortic root expansion in MFS patients [167, 169, 172]. This is particularly interesting as the usage of β-blockers, the current mainstay of treatment, is predominantly based on the results of a single clinical trial, published in 1994 [173]. This trial had many limitations, such as a very limited sample size, unblinded treatment allocation, a lack of control on drug compliance and inclusion of deaths in the analyses that were unrelated to aortic pathology [174]. Also, a recent Cochrane review stated that no clear treatment recommendation regarding the use of β-blockers in MFS could be made [175]. Therefore, solid evidence of the beneficial effects of β-blockers in MFS patients is lacking [176]. Notably, none of the performed trials reported superiority of β-blockers over ARBs. The largest among them, the Pediatric Heart Network Trial [167], compared losartan with the β-blocker atenolol in 608 young MFS patients. After 3 years of follow-up, both drugs were equally effective in attenuating aortic root dilatation. These results were verified by a Spanish randomized controlled trial with a similar design [169]. Nonetheless, a recent review [177] suggests that caution concerning ARBs as first-line monotherapy is warranted, as adverse events were almost twice as common in the losartan group compared to the atenolol group in the Pediatric Heart Network Trial. However, in the Spanish randomized controlled trial, only 1 serious adverse event occurred in the losartan group, compared with 4 in the atenolol group [169]. Importantly, neither trials reached statistical significance regarding these adverse endpoints [167, 169]. In addition, an extension study of the Spanish trial by Forteza et al. found no differences in adverse outcomes in the losartan vs. the atenolol group after a median follow-up period of 6.7 years [178]. Thus, ARBs do not seem to be inferior to β-blockers in attenuating aortic root growth in MFS patients and display a similar long-term safety profile.

### ARBs and β-Blockers: Synergy?

Interestingly, other studies examined potential synergistic effects of combining a β-blocker with an ARB in syndromic TAAs. Although a synergy between β-blockers and ARBs was observed in a small, open-label randomized controlled trial [166], the beneficial effects of add-on losartan therapy were not confirmed by the Ghent Marfan Trial [170]. Perhaps this study was underpowered, but the much larger French Marfan Sartan trial also failed to confirm this synergy [168]. In this latter study, 86% of the 303 included MFS patients used β-blocker therapy at baseline. After a median follow-up of 3.5 years, the addition of 50–100 mg losartan did not result in a slower dilatation of the aortic root compared with placebo. Notably, this lack of synergy between ARBs and β-blockers was challenged by the COMPARE trial [129] and, more recently, by the multicenter randomized controlled AIMS trial [130]. The AIMS trial demonstrated that addition of the ARB irbesartan to usual care significantly decreased aortic root growth compared to placebo. In this add-on irbesartan group, 54% used concomitant β-blocker therapy and mean blood pressure levels were slightly lower, which could contribute to the observed differences.

Notably, all randomized clinical trials examined aortic dilatation, but none had sufficient statistical power to detect any differences in the clinically most relevant outcomes, such as aortic dissection, aortic rupture and mortality. Therefore, the results from the recently published long-term follow-up study of the COMPARE trial are highly relevant [179]. After a median follow-up period of 8 years, a clear reduction in several adverse clinical endpoints, such as all-cause mortality and aortic dissection, was observed in the patient group that used losartan during the entire follow-up period. Interestingly, 81% of these patients used concomitant β-blockade therapy. In addition, a recent meta-analysis demonstrated that the addition of an ARB to β-blocker therapy reduced the rate of aortic root dilatation, but did not significantly reduce the number of aortic complications [180]. This latter result could be a consequence of low statistical power to demonstrate differences in these complications due to low event rates. Also, this meta-analysis did not include the long-term follow-up study from the COMPARE trial [179]. Thus, when taking the results from the COMPARE, AIMS, and the long-term follow-up study of the COMPARE trial together, it seems that combining ARBs and β-blockers in MFS patients is not only preferential to prevent aneurysm growth but also to prevent long-term adverse outcomes [129, 130, 179].

### Differences Between Preclinical and Clinical Studies

Unfortunately, the promising preclinical results of ARBs to attenuate TAA expansion [37, 106] were not unequivocally reproduced in the clinical setting and the observed discrepancy was remarkable. Several reasons could underlie the seemingly lack of consistent results in the human situation. Firstly, the efficacy of ARB treatment might be determined by the type and dosage of ARB used. The AIMS trial demonstrated that addition of irbesartan to usual care was beneficial, whereas losartan did not lead to superior treatment outcomes in the Marfan Sartan Trial. As irbesartan has a much longer half-life and a greater bioavailability than losartan [181], this could indicate that losartan dosages in previous studies were insufficient to fully benefit from its protective effects. Indeed, atenolol was administered in much higher dosages than losartan in the Pediatric Heart Network Trial which led the authors to conclude that a higher dose of losartan or a different ARB might yield superior results [167]. Previously, it has been suggested that administered dosages of RAS inhibitors, including ARBs, in the range to reduce blood pressure are insufficient to effectively normalize TGF-β overexpression [182]. Provided that these higher ARB dosages are well-tolerated by the included patients, it would be interesting to study if these higher dosages can attenuate aortic dilatation more effectively.

Secondly, it could be essential to start ARB treatment early in aneurysm development. We hypothesize that elevated aortic Ang II-AT1R signaling is an important driver for excessive TGF-β signaling in the context of syndromic TAAs. Consequently, early specific inhibition of Ang II-AT1R signaling is necessary to prevent this detrimental TGF-β upregulation, but still allowing for RAS-independent basal TGF-β signaling to occur. Indeed, a preclinical study demonstrated that losartan prevented aneurysm formation in a VSMC-specific *Fbln4* knock-out mouse model when initiated from postnatal day 7, whereas this treatment lost its efficacy when initiated at a later time point [100]. Also, in the hallmark trial showing the beneficial effect of losartan in *Fbn1*^*C1041G/+*^ mice, this treatment was started prenatally [106]. How this critical treatment time window can be translated to the human situation needs further investigation. Also, determinants other than age, such as aortic root Z-scores, may be better indicators when to initiate medical treatment, as they better reflect aortic disease stage. In addition, higher circulating TGF-β levels are associated with more advanced stages and higher rates of aortic dilatation in TAA patients [149]. In this regard, it would be interesting to investigate if circulating TGF-β levels could be used as a biomarker for the severity of aortic pathology, in concordance with other possible biomarkers [183]. Of note, factors other than Ang II can also directly stimulate the AT1R in syndromic TAAs, such as mechanical stress due to disturbed mechanosensing. Therefore, specific direct inhibition of AT1R signaling via ARBs, rather than inhibition of Ang II via ACEi, is supposed to yield the most optimal treatment outcomes, although a direct comparison has not been made in syndromic TAA patients.

Thirdly, results from preclinical studies are based on a limited number of mouse models, harboring identical mutations in the *Fbn1* gene (Table 1) [14]. In contrast, more than 1000 mutations in *FBN1* have been identified in human MFS patients [184, 185]. Therefore, these preclinical studies do not adequately represent the genetic heterogeneity found in MFS patients and this could hamper the translation of promising preclinical results into the clinical situation.

Fourthly, this genetic heterogeneity in MFS patients can also be an underlying cause for the differences in treatment outcomes observed between clinical trials. Most clinical studies did not assess nor stratify for the underlying *FBN1* mutation. As a consequence, these mutations might not be equally distributed among treatment groups and between different studies. Of note, characterizing the underlying *FBN1* mutation is extremely relevant, as the type of mutation determines the clinical course and severity of aortic pathology in MFS patients [186, 187]. Also, it predicts the efficacy of losartan therapy: in a substudy of the COMPARE trial, MFS patients carrying a haploinsufficient *FBN1* mutation responded better to losartan therapy than those harboring a dominant-negative mutation [188]. Therefore, differences in underlying *FBN1* mutations could be another reason for the observed variability of treatment effects in clinical trials.

To summarize, multiple factors seem to underlie the observed differences between the preclinical and clinical situation. Therefore, the results from the announced prospective meta-analysis, combining all previous randomized controlled trials on ARB and β-blocker treatment in MFS, are eagerly awaited [189]. Hopefully, this meta-analysis will clarify the therapeutic potential of these compounds in different MFS subgroups, such as clustered by age, aortic Z-score, or type of *FBN1*-mutation, on the most relevant clinical outcomes.

## Conclusions and Future Directions

Over the past decades, a better understanding of the molecular mechanisms leading to the formation of syndromic TAAs has been obtained. Elevated signaling of the TGF-β pathway is observed in syndromic TAA patients, which could be due to increased local RAS signaling. These pathways are likely to act in concert to induce aortic pathology by mechanisms that remain subject of investigation, but at least include stimulation of aortic ECM degradation and aortic fibrosis. However, how elevated RAS signaling occurs in the context of TAAs is yet to be fully elucidated. Mechanical stress resulting from disruption in mechanosensing abilities of aortic VSMCs might be one of the contributors. Importantly, preclinical evidence suggests that targeting the TGF-β pathway too early in aortic aneurysm formation leads to worsening of aortic pathology. Thus, clinical treatment in affected TAA patients should ideally be directed at both RAS and TGF-β signaling, although sparing the early protective role of the latter. In this regard, biomarkers indicating the stage of aortic disease could be of additional value. Also, higher ARB dosages combined with therapeutic modalities that reduce mechanical stress, such as β-blockers, might be preferential. As ERK1/2 also seems to play a crucial role, an alternative treatment strategy might be to specifically inhibit ERK1/2-signaling in syndromic TAAs patients. Such drugs are currently being investigated in the field of oncology [190].

Importantly, to the best of our knowledge, all clinical trials investigating the efficacy of ARBs and β-blockers on aortic pathology were performed in MFS individuals. Unfortunately, these clinical trials did not confirm the promising results from preclinical studies. Several reasons could underlie these differences, including wide genetic heterogeneity among the MFS population, which affects disease severity and treatment efficacy. Hopefully, future research will take these genetic aspects into account. A difficulty to overcome is the fact that only a limited number of patients for each specific mutation are available. Also in this light, caution is warranted regarding the extrapolation of results from the MFS study population to patients with other TAA syndromes, such as LDS, and more research is needed in these individual patients and patient populations. In this regard, bioinformatic predictions on the consequences of different mutations might be useful to form more specific, but large enough study groups and investigate treatment outcomes in these subgroups. This knowledge can be combined with new developments in induced pluripotent stem cells technology, for which patient material can be used to improve and accelerate the translation of preclinical findings into the clinical situation [191]. Combining these techniques with new integrative approaches coupling genomics, transcriptomics, proteomics, and functional experiments [62, 192] could pave the way for novel targeted and more individualized treatment regimens for syndromic TAA patients.

## Data Availability

PubMed, Embase, Web of Science, and Cochrane Library search.
